# Screening of reference genes in real-time PCR for *Radopholus similis*

**DOI:** 10.7717/peerj.6253

**Published:** 2019-01-16

**Authors:** Jun-Yi Li, Wan-Zhu Chen, Si-Hua Yang, Chun-Ling Xu, Xin Huang, Chun Chen, Hui Xie

**Affiliations:** Laboratory of Plant Nematology and Research Center of Nematodes of Plant Quarantine, Department of Plant Pathology, Guangdong Province Key Laboratory of Microbial Signals and Disease Control, College of Agriculture, South China Agricultural University, Guangzhou, Guangdong Province, People’s Republic of China

**Keywords:** Radopholus similis, Reference gene, Real-time PCR

## Abstract

Six candidate reference genes were chosen from the transcriptome database of *Radopholus similis* using the bioinformatics method, including four conventional reference genes * (actin*, Eukaryotic translation initiation factor 5A (*eIF5A*), Tubulin alpha (*a-tubulin)*, ubiquitin (*UBI*)) and two new candidate reference genes (Ribosomal protein S21 (*Rps21*) and Serine/threonine protein phosphatase PP1-β catalytic subunit (*β*-PP1)). In addition, a traditional reference gene 18S ribosomal RNA (*18S rRNA*) obtained from NCBI databases was also added to the analysis. Real-time PCR was used to detect the expression of seven candidate reference genes in six populations of *R. similis* and four developmental stages (female, male, larva and egg) of a population. The stability of the expression of candidate genes was evaluated by three software programs, BestKeeper, geNorm and NormFinder. The results showed that *eIF5A* is the most suitable reference gene for gene functional research of different populations, while both *Rps21* and *eIF5A* are the most suitable reference genes for different developmental stages of a population. Therefore, *eIF5A* is the best reference gene for studying *R. similis*. However, one defect of this study is that only seven candidate reference genes were analyzed; ideally, more genes should be tested.

## Introduction

Polymerase chain reaction (PCR) is a technique that can amplify certain DNA fragments in vitro (Saiki et al., 1985). Real-time quantitative polymerase chain reaction (Real-time PCR), a modified PCR technique that adds a fluorescent dye or fluorescent probe to the reaction system during the PCR reaction, enables quantitative analysis of an initial template that is positively correlated with its product by real-time monitoring of fluorescence signal strength changes, achieving real-time detection of each round of PCR reaction products (Ginzinger & David, 2002; Schmittgen, 2001). Compared with end-point quantitation of ordinary PCR techniques, real-time-PCR is superior in terms of accuracy, repeatability, specificity, sensitivity and convenient operation; therefore, it has been widely used in various fields of molecular biology (Bustin & Dorudi, 1998; Gachon, Mingam & Charrier, 2004; Higuchi et al., 1993).

In general, real-time PCR can be divided into absolute quantification PCR and relative quantification PCR. Absolute quantification PCR requires the preparation of a standard sample with a known concentration that needs to be diluted before the PCR reaction and the drawing of a standard curve (Bustin, 2000). However, in many cases, we only need to determine the relative differences in gene expression without the need for absolute quantification; in this situation, using relative quantification PCR is a more general and simpler method. This method does not require a standard sample but instead calculates the sample changes in the amount of target gene relative to a reference gene that should be constantly expressed in body cells. Therefore, the authenticity of the relative quantification results must be based on a reliable reference gene (Bustin, 2002; Suzuki, Higgins & Crawford, 2000). The ideal reference genes should be stably expressed in various cells, tissues, and organs; different populations; different developmental stages; different cell cycle stages; and different treatment conditions. However, studies have shown that the expression of many commonly used reference genes is not absolutely constant; so-called constant expression is only relatively constant under certain treatment conditions or in certain types of tissues (Andersen, Jensen & Orntoft, 2004; Liu, Chen & Liu, 2005; Radonić et al., 2004). Reference genes have no general applicability, which means that they may result in inaccurate quantitative results if one or several reference genes are randomly selected (Arya et al., 2005; Wong & Medrano, 2005). Therefore, for different research objects, we need to consider different factors and select the most suitable reference gene by analyzing whether the candidate reference gene is expressed constantly or not (Thellin et al., 1999).

The current software for the analysis of reference gene stability includes BestKeeper (Pfaffl et al., 2004), geNorm (Vandesompele et al., 2002) and NormFinder (Andersen, Jensen & Orntoft, 2004). BestKeeper calculates the standard deviation (SD), coefficient of variance (CV) and coefficient of correlation (*r*) of candidate reference genes, a candidate reference gene with a small SD value and a small CV value but a large *r* value is selected as an appropriate reference gene (Pfaffl et al., 2004). geNorm can calculate the stable value *M* of each candidate reference gene, and a smaller M value indicates more stable expression of the gene. The default *M* value is 1.5; when *M* < 1.5, it can be considered a suitable reference gene. The software also introduces a pairwise variation value V to determine the optimal number and best combination of reference genes with a default value of 0.15. When *V*_*n*∕*n*+1_ < 0.15, the *n* + 1 candidate reference genes do not need to be introduced. When *V*_*n*∕*n*+1_ > 0.15, the *n* + 1 candidate reference gene need to be introduced until *V*_*n*∕*n*+1_ < 0.15. However, the default value 0.15 may not meet the requirements in some experiments. Therefore, the corresponding changes should be made in combination with the experimental results (Vandesompele et al., 2002). The calculation principle of NormFinder is similar to that of the geNorm software, which means the most suitable reference gene is screened according to the stability value calculated by the software (Andersen, Jensen & Orntoft, 2004; Susana, Isabel & Raquel, 2008).

There are many scientific reports of reference gene screening for different organisms in recent years (Galli et al., 2015; Libault et al., 2008; Tong et al., 2009; Trivedi & Arasu, 2005), including some reports on free-living nematodes and animal parasitic nematodes (Hoogewijs et al., 2008; Lecová et al., 2015). However, the screening of plant parasitic nematode reference genes is rarely reported. The burrowing nematode *Radopholus similis* is a migratory endoparasitic plant nematode that is extremely devastating and is one of the 10 most common plant nematodes in the world (Jones et al., 2013). To explore ways to use molecular biology techniques to effectively control *R. similis*, some recent research has been carried out on pathogenicity genes of *R. similis* (Haegeman et al., 2010a; Haegeman, Vanholme & Gheysen, 2010b; Huang et al., 2017; Jacob et al., 2008; Jacob et al., 2007; Ke et al., 2016; Li et al., 2015a; Li et al., 2015b; Zhang et al., 2015; Zhang et al., 2012). When investigating the pathogenesis and developmental expression patterns of these genes or evaluating the silencing effect of RNAi on them, the researchers mostly chose either *actin* or *18S rRNA* as a reference gene; however, the stability of these two reference genes has not been studied in depth. Screening and identification of reference genes based on transcriptome sequencing in real-time PCR has been an effective strategy in recent years. Cankorur-Cetinkaya (Cankorurcetinkaya et al., 2012) applied this strategy to identify a new set of reference genes in yeast and the same strategies have also been applied to filamentous fungi (Vieira et al., 2016), plants (Chang et al., 2012) and animals (Hu, Xie & Yao, 2016; Zhan et al., 2014) . In our previous work, in order to understand the developmental, reproductive and parasitic characteristics of *R. similis* at the molecular level, we have generated transcriptome data from different developmental stages of *R. similis* (accession number: SRR6425985 –SRR6425988) by *de novo* sequencing with the Illumina HiSeqTM 2000 platform. In this study, seven candidate reference genes, including *actin* and *18S rRNA* extracted from transcriptome data of *R. similis* or obtained from NCBI databases, were analyzed for expression stability in six populations of *R. similis* and four developmental stages of a population. The results show that both *actin* and *18S* rRNA are not the most stable reference genes, while *eIF5A* is the best reference gene for *R. similis*.

## Materials & Methods

### Nematode

The experimental populations of *R. similis* were isolated and identified by the Plant Nematode Research Laboratory of South China Agricultural University and then cultured and preserved on the callus of carrot (*Daucus carota* L.) according to a reported method (Fallas & Sarah, 1995) at 25 ± 1 °C in the dark (0-h light/24-h dark photoperiod).

Four developmental stages (female, male, larva and egg) of the *R. similis* GJ population were collected (approximately 3,000 individuals each), sterilized with 0.3% streptomycin sulfate for 6 h, washed three times with DEPC water and finally pipetted to remove water; approximately 3,000 mixed-stage nematodes of six populations (Table 1) were collected, and the subsequent treatments were the same as above. All samples were placed in liquid nitrogen and frozen at −80 °C for later use.

**Table 1:** Origin and host of *Radopholus similis* populations used in the study.

Population code	Host
dbsr	*Anubias barteri* var.barteri
GJ	*Citrus reticulata* Blanco
HaiN-H	*Anthurium andraeanum*
HN6	*Musa* AAA Giant Cavendish cv.Baxi
Xin	*Zingiber officinale*
YJ	*Radix curcumae*

**Table 2:** The basic information of seven candidate reference genes of *Radopholus similis*.

Genes for short	Full name of genes	Source of genes
*actin*	actin	Transcriptome database (accession number: SRR6425985–SRR6425988) preserved by our group
*Rps21*	Ribosomal protein S21	
*eIF5A*	Eukaryotic translation initiation factor 5A	
*a-tubulin*	Tubulin alpha	
*UBI*	Ubiquitin protein	
*β-PP1*	serine/threonine protein phosphatase PP1-beta catalytic subunit	
*18S rRNA*	18S ribosomal RNA	NCBI (AJ966502.1)

### Total RNA extraction and synthesis of cDNA

Total RNA of mixed-stage nematodes from six populations of *R. similis* and four developmental stages of the GJ population were extracted according to the instructions of HiPure Total RNA Plus Kits (Magen, Guangzhou, Guangdong, China). Total RNA concentration and purity were determined by a NanoDrop 2,000 (Thermo Fisher, Waltham, MA, USA) nucleic acid analyzer, while RNA integrity was detected by 1% agarose gel electrophoresis. Qualified RNA was reverse transcribed to synthesize cDNA using a ReverTra Ace qPCR RT Kit (TOYOBO, Shanghai, China), and the resulting cDNA was stored at −20 °C.

### Extraction and cloning analysis of candidate reference genes

Relatively stably expressed transcripts were selected from the transcriptome of *R. similis* satisfying the conditions —log2Ratio— ≤ 1 and FDR ≤ 10^−10^ and analyzed by performing a BLAST search of the NCBI nonredundant protein database (nr) to obtain the annotation information. Six transcripts annotated as *actin*, *Rps21*, *eIF5A*, *a-tubulin*, *UBI* and *β-PP1* from transcriptome data of *R. similis* (accession number: SRR6425985 –SRR6425988) were found to meet the requirements of the experiment. Therefore, these six genes of *R. similis* were used as candidate reference genes, and *18S rRNA* extracted from NCBI databases (accession number AJ966502.1) was also used as a candidate reference gene (Table 2). Among them, *actin*, *a-tubulin*, *UBI*, *eIF5A* and *18S rRNA* are the traditional reference genes, while *Rps21* and *β-PP1* are newly identified ones. According to the transcript sequences of six candidate reference genes in the transcriptome data of *R. similis,* Primer Premier 5.0 (Lalitha, 2000) was used to design the specific primers required for PCR amplification with the mixed-stage cDNA of the GJ population as a template (Table 3). PCR products were detected by 1% agarose gel electrophoresis, followed by recovery of the target fragment using a Gel Pure DNA Mini Kit (Magen, Guangzhou, Guangdong, China). The recovered PCR products were sequenced, and the resulting sequences were used to perform a BLAST search of the NCBI nonredundant protein database (nr) to acquire information on homology alignment and similarity.

**Table 3:** The primer sequences for cloning six candidate reference genes of *Radopholus similis*.

Gene	Primer sequences(5′–3)	Product size (bp)
*actin*	F:GGGCGTAACCCTCGTAGATG R:ATGGTCGGAATGGGACAGAA	382
*Rps21*	F:ACCCAGTACTCAAGGTCAAA R:AGAAACAGTCAACAAATCGC	344
*eIF5A*	F: GCCGCTGCCACTTACCCGAAACA R:TTGGACGAGAGAGAGGAGAA	556
*a-tubulin*	F:ATCACCGCATCTCTCCGCTT R:CGCCTTCCTCCATTCCCTCT	538
*UBI*	F:CGTGAAAACTCTGACTGGAAAG R:CCTCTGCGCTTTCTCCCATT	473
*β-PP1*	F: CTTCTGCTGTCATGGCGGACTTTCG R: GACGGTCGTAGTGCTGCTAACCTTTCAA	719

**Table 4:** The primer sequences and amplification parameters of seven candidate reference genes of *Radopholus similis* used in qPCR analysis.

Gene	Primer sequences(5′–3)	Product size (bp)	Amplification efficiency (%)	*R*^2^
*actin*	F:CTCGTTGTAGAAGGTGTG R:CTGAAGTACCCGATTGAG	81	93.68	0.9957
*Rps21*	F:TGGCACAGAAAGATGGAAT R:AACAGTCAACAAATCGCAAT	76	97.40	0.9991
*eIF5A*	F:AGAGACGAGGATGAGTTT R:GAGAGAGGAGAATTTGTTGAT	83	92.11	0.9993
*a-tubulin*	F:TCAACTACCAGCCGCCAACT R:CCTTCCTCCATTCCCTCTCC	184	95.71	0.9948
*UBI*	F:CTTCGTCAAGACCCTCAC R:ATCTTCGCTTTCACATTCTC	81	100.27	0.9958
*β-PP1*	F:CGACGGTAAAGAAACATTA R:GCCTACTTGCTTAAACTG	134	93.87	0.9951
*18S rRNA*	F:CACAAAAACTCCCAACGCAA R:ATTCAACACTCAACCCCCGA	78	96.85	0.9979

### QPCR of candidate reference genes and calculation of amplification efficiency

Before running qPCR, primers designed by Primer Premier 5 for qPCR of seven candidate reference genes were tested by ordinary PCR with the mixed-stage cDNA of the GJ population as a template to verify whether they are specific and whether dimers exist. PCR products were detected by 1% agarose gel electrophoresis.

The cDNAs of mixed-stage nematodes of six populations (dbsr, GJ, HaiN-H, HN6, Xin, and YJ) and four developmental stages of the GJ population (female, male, larva and egg) were used as templates in the qPCR using the designed primers (Table 4), each reaction was set up with three biological replicates and three technical replicates. The total volume of the qPCR system was 20 µl: cDNA, 2 µl; sense/antisense primer, 1 µl each; SYBR Green Master Mix (Vazyme, Nanjing, Jiangsu, China), 10 µl; and ddH_2_O, 6 µl. qPCR was performed in a two-step method using a CFX96 qPCR instrument (Bio-Rad, Hercules, CA, USA). The qPCR conditions were as follows: predenaturation at 95 °C for 5 min, denaturation at 95 °C for 10 s, and annealing and extension at 60 °C for 60 s; fluorescence signals were collected during annealing and extension and the whole process was repeated for 40 cycles. Melting curve analysis was performed at the end of the amplification from 65 °C to 95 °C and with a hold of 0.05 s every 0.5 °C. Additionally, the cDNA of the mixed-stage GJ population was designated a standard sample and successively diluted by a factor of 5 with Easy Dilution to create seven concentration gradients in the order of 5^−1^, 5^−2^, 5^−3^, 5^−4^, 5^−5^, 5^−6^, and 5^−7^ of the initial template. A standard curve was drawn based on the logarithm of the relative cDNA concentration of the template to be the abscissa and the corresponding Ct value to be the ordinate, then the slope of the standard curve was obtained and the amplification efficiency (*E*) of candidate reference genes was calculated according to the formula *E* = [10(−1∕slope) − 1] × 100% (Livak & Schmittgen, 2001).

### Stability analysis of candidate reference genes

The original cycle thresholds (Ct values) of seven candidate reference genes were obtained from Bio-Rad CFX-96 Manager, and the data were sorted by Excel to evaluate the differences in the expression levels of seven candidate reference genes. Subsequently, the stability analysis of candidate reference genes was carried out by three software packages, including BestKeeper v1 (https://www.gene-quantification.de/bestkeeper.html#download), geNorm embedded in qBasePlus (https://genorm.cmgg.be/) and NormFinder v20 (https://www.moma.dk/normfinder-software/). The results of the three software packages were then compared and further analyzed to determine which gene is the most suitable reference gene.

## Results

### Cloning and analysis of candidate reference genes

**Figure 1:**
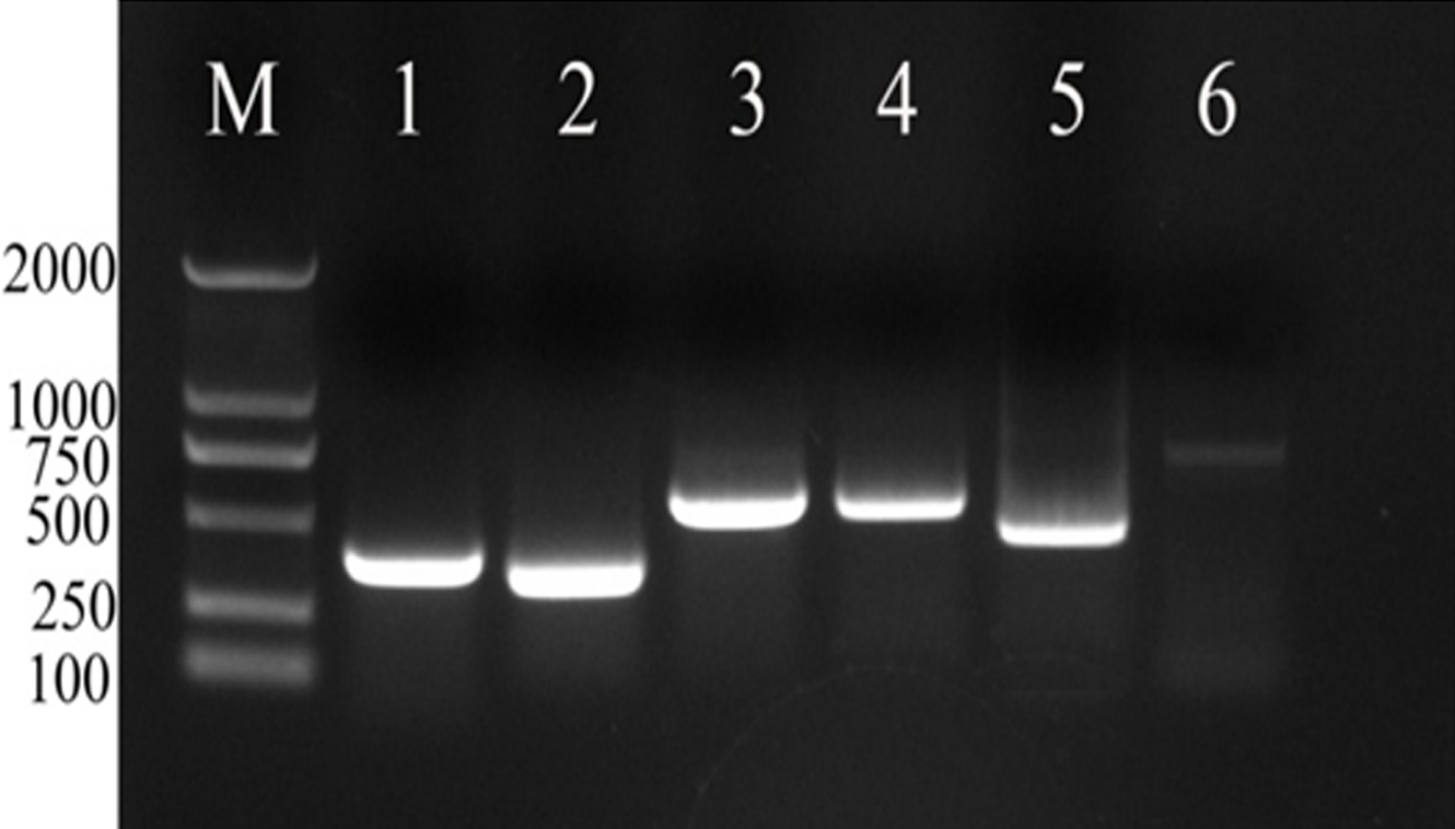
PCR analysis of positive clones of candidate reference genes of *Radopholus similis*.

**Figure 2:**
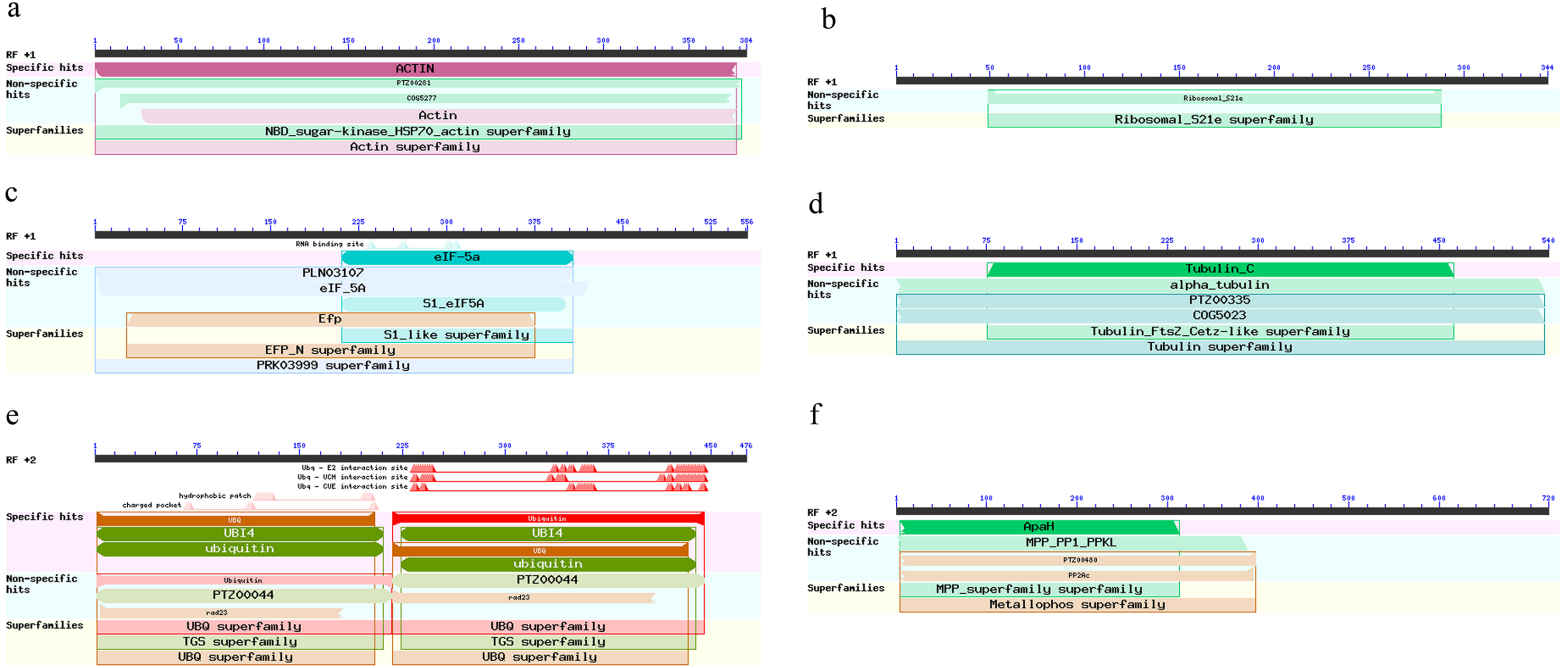
Prediction of conserved domains of six candidate reference genes of *Radopholus similis*. (A–F) Gene fragments of *actin*, *Rps21*, *eIF5A*, *a-tubulin*, *UBI* and * β-PP1* of *R. similis*.

The PCR amplification fragments of candidate reference genes *actin*, *Rps21*, *eIF5A*, *a-tubulin*, *UBI* and *β-PP1* from *R. similis* are 382 bp, 344 bp, 556 bp, 538 bp, 473 bp and 719 bp, respectively (Fig. 1), and all are consistent with the expected size and the corresponding sequence of these genes from the transcriptome of *R. similis*. The sequences of the fragments mentioned above were used to perform a BLAST search of the NCBI nonredundant protein database (nr); the results showed that the amino acid sequences are highly similar between the six candidate reference gene fragments and the corresponding genes of other nematodes (Table 5). Further analysis revealed that these cloned fragments all have conserved domains of the proteins encoded by the corresponding genes (Fig. 2). Therefore, we can confirm that the cloned gene fragments are *actin*, *Rps21*, *eIF5A*, *a-tubulin*, *UBI* and *β-PP1* gene fragments of *R. similis*. We then uploaded these sequences to Genebank and got the corresponding accession number (MH499256, MH499257, MH499258, MH499259, MH499260, MH499261).

**Table 5:** The amino acid similarity between the predicted amino acid sequences of seven candidate genes of *Radopholus similis* and that of corresponding genes of other nematodes.

	Fragment length (bp)	*Teladorsagia circumcincta*	*Wuchereria bancrofti*	*Caenorhabditis elegans*	*Brugia malayi*	*Toxocaracanis*	*Loa loa*	*Strongyloides ratti*
*actin*	382	99%	99%	100%	99%	98%	–	99%
*Rps21*	344	70%	70%	77%	70%	71%	72%	70%
*eIF5A*	555	78%	84%	77%	85%	83%	85%	82%
*a-tublin*	538	–	86%	89%	92%	93%	93%	89%
*UBI*	473	97%	97%	–	95%	97%	97%	87%
*β-PP1*	719	–	89%	95%	91%	92%	92%	87%

### qPCR primer specificity and reliability analysis

**Figure 3:**
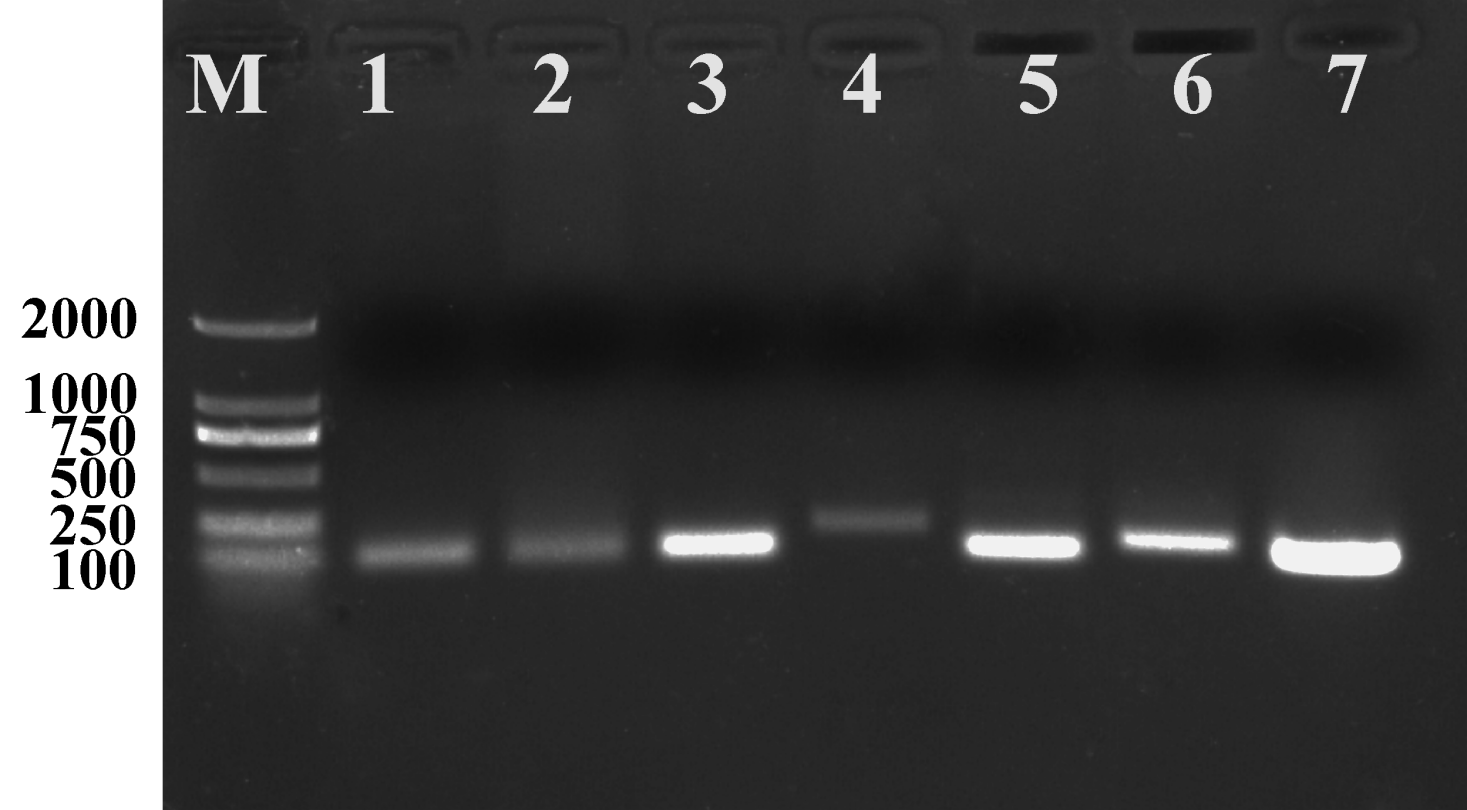
PCR products of seven candidate reference genes of *Radopholus similis* amplified by qPCR primers.

**Figure 4:**
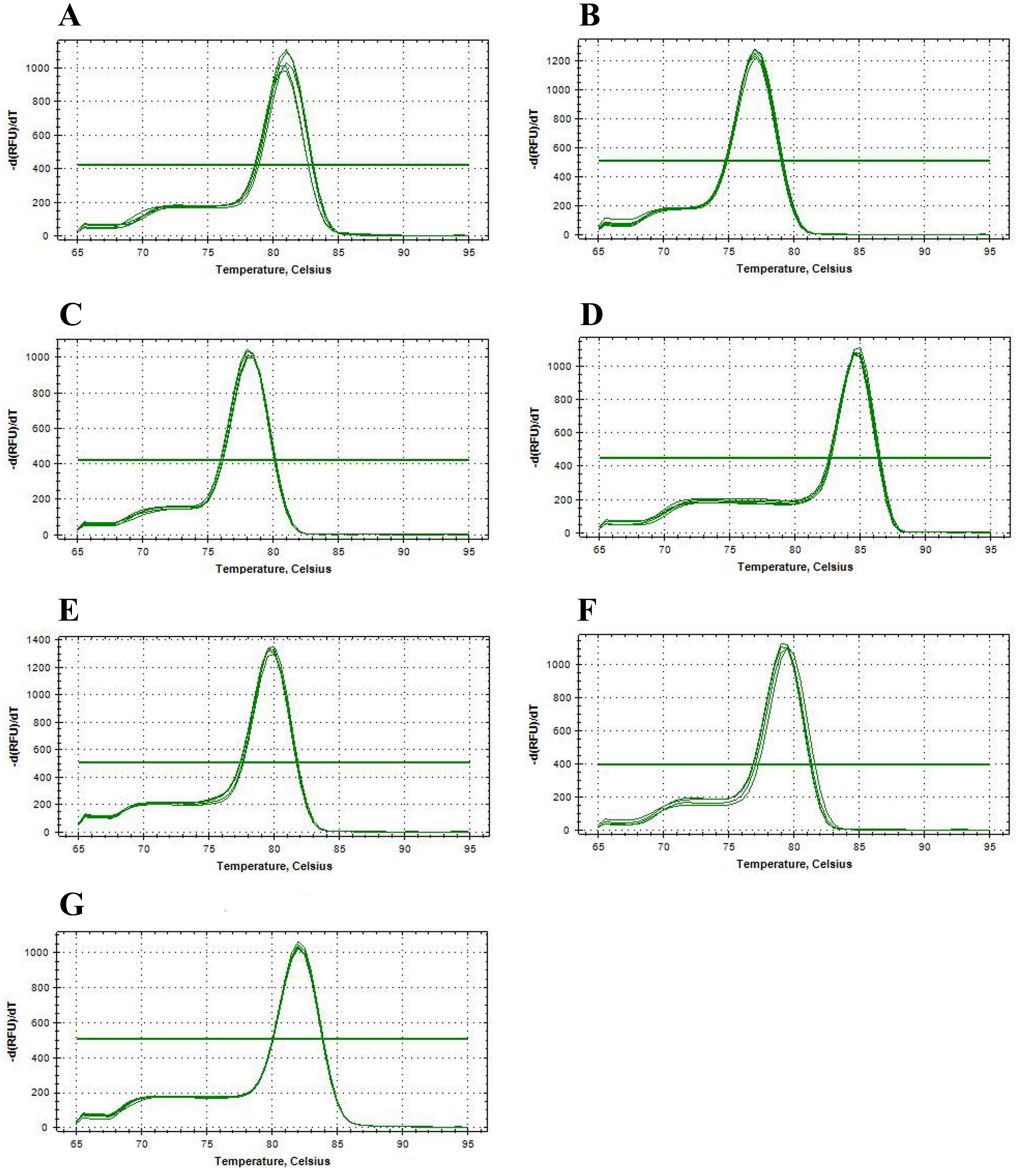
Melting curve analyses of seven candidate reference genes of *Radopholus similis* for qPCR. (A–G) Melting curves of *actin*, *Rps21*, *eIF5A*, *a-tubulin*, *UBI*, * β -PP1* and *18SrRNA* of *R. similis*.

**Figure 5:**
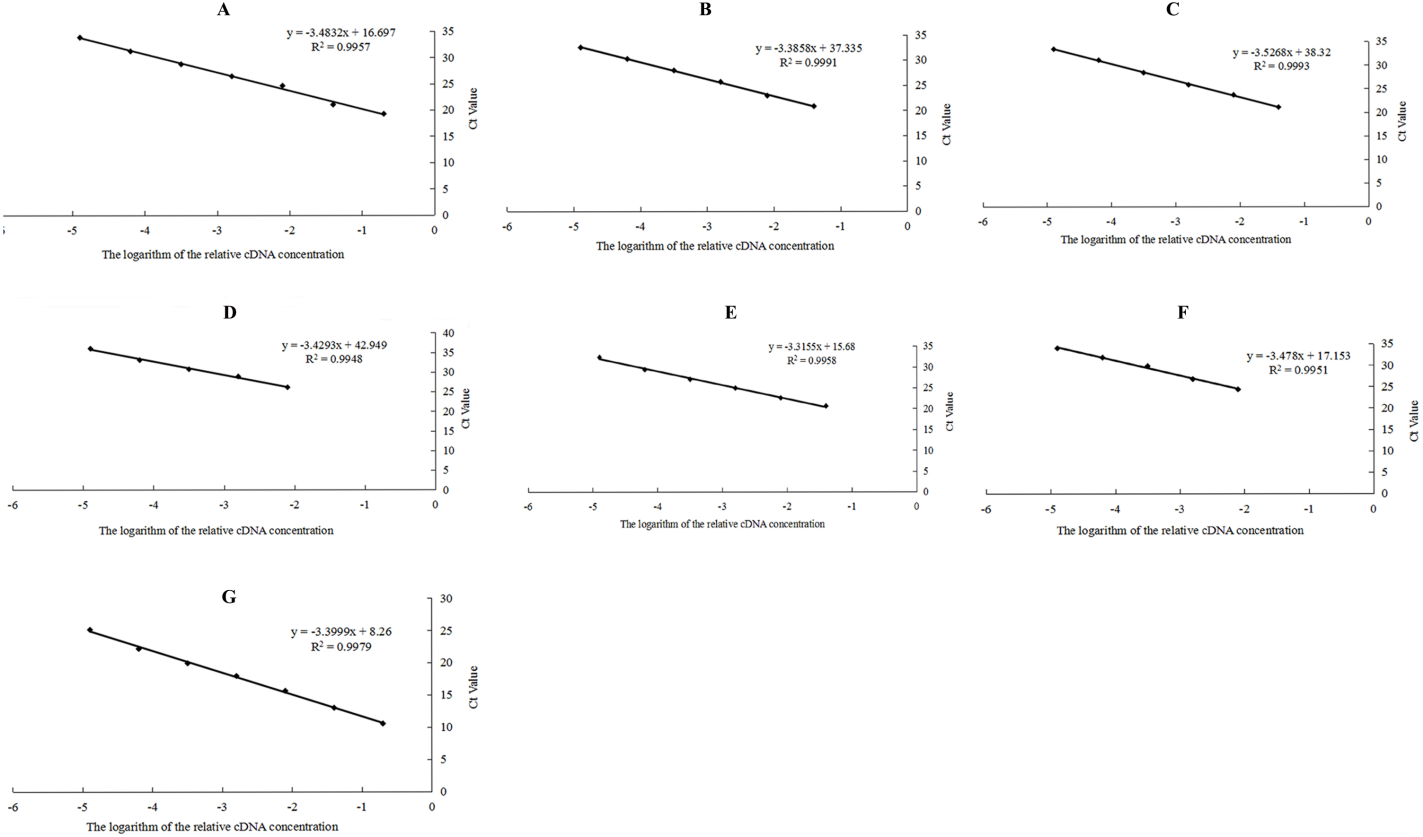
Standard curves of seven candidate reference genes of *Radopholus similis*. (A–G) Standard curves of *actin*, *Rps21*, *eIF5A*, *a-tubulin*, *UBI*, * β -PP1* and *18SrRNA* of *R. similis*.

The qPCR primers of candidate reference genes were subjected to specific analysis by ordinary PCR prior to performing qPCR. The results showed that the seven candidate reference genes produced a single band (Fig. 3) and no primer dimers, indicating that the primer specificity was good. The seven candidate reference genes were amplified by qPCR using the cDNA of the mixed-stage nematode of the GJ population as a template. The melting curves of the seven candidate reference genes are all single peaks (Fig. 4), indicating that the specificity of the primers is good and no primer dimers are present. In addition, standard curves (Fig. 5) were drawn to calculate the amplification efficiency (E) and correlation coefficients (R^2^) according to the Ct values of each candidate reference gene amplified by qPCR using the cDNA of the mixed-stage nematode of the GJ population as a standard sample. The results showed that the *E* value of each candidate reference gene is between 90–110% (Table 4), and the R^2^ value is greater than 0.99, which means the data are highly credible and can be used for data analysis.

**Figure 6:**
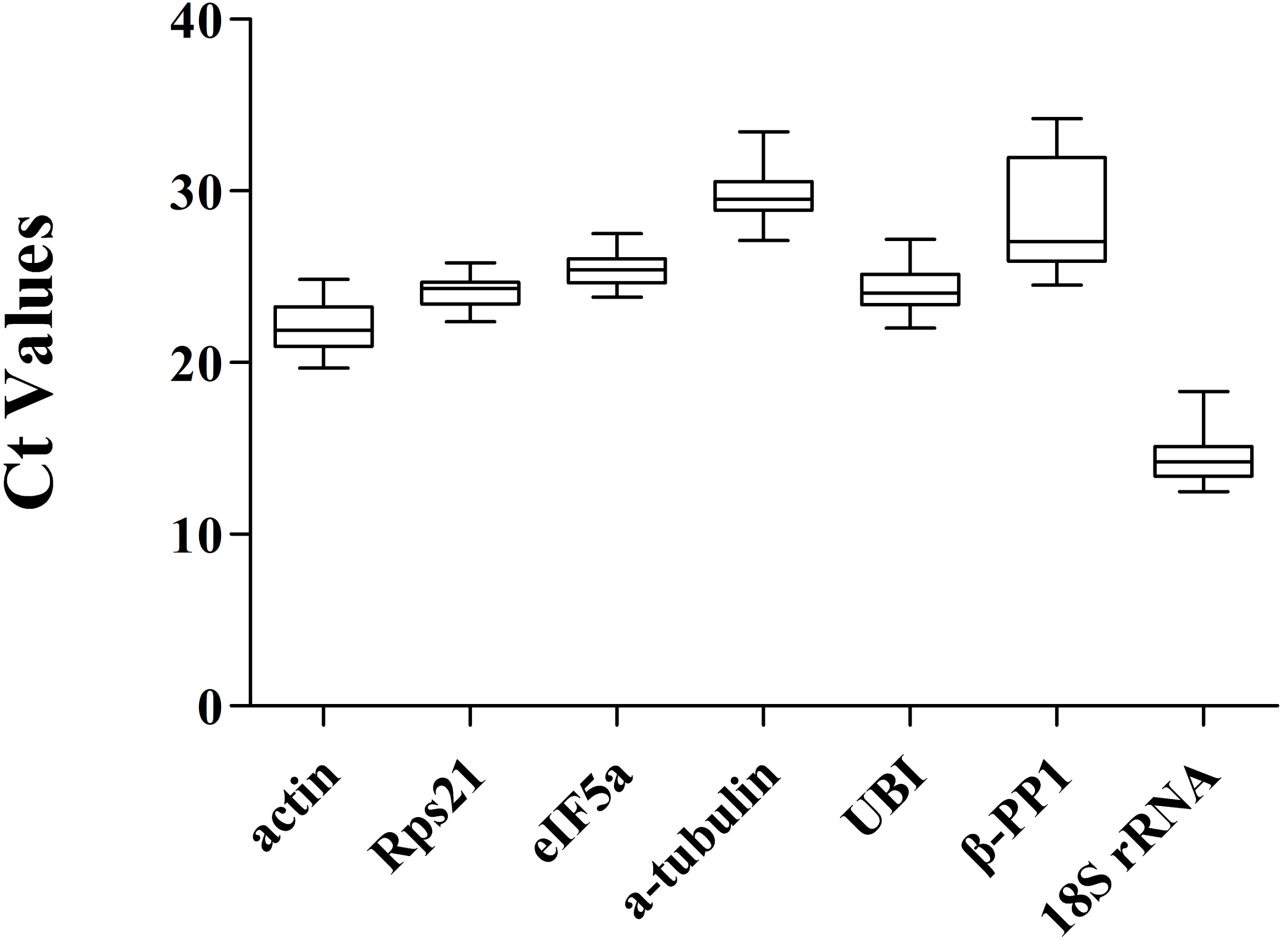
Range of all Ct values of seven candidate reference genes under two experimental conditions of *Radopholus similis*. (A) mixed-stage nematodes of six populations of *R. similis* isolated from *Anubias barteri* var. barteri, *Citrus reticulata* Blanco, *Anthurium andraeanum, Musa* AAA Giant Cavendish cv. Baxi, *Zingiber officinale* and *Radix curcumae*. (B) Four developmental stages including female, male, larva and egg of the *R. similis* GJ population isolated from *Citrus reticulata* Blanco.

### qPCR expression analysis of candidate reference genes

The distribution of the qPCR Ct values of the seven candidate reference genes (Fig. 6) in six populations of *R. similis* and in four developmental stages of the GJ population showed that the Ct values of each candidate reference gene fluctuated in the range of 12.47 to 34.22. The Ct values of *actin*, *Rps21*, *eIF5A*, *a-tubulin*, *UBI*, *β-PP1* and *18S rRNA* ranged from 19.69∼24.86, 22.39∼25.81, 23.82∼27.50, 27.13∼33.45, 22.02∼27.19, 24.53∼34.22 and 12.47∼18.31, respectively, of which *Rps21* and *eIF5A* fluctuated the least while *β-PP1* fluctuated the most. The difference between the maximum and minimum of each candidate reference gene Ct values (d) in ascending order is *Rps21* (*d* = 3.42), *eIF5A* (*d* = 3.68), *actin* (*d* = 5.17), *UBI* (*d* = 5.17), *18S rRNA* (*d* = 5.84), *a-tubulin* (*d* = 3.42), and *β-PP1* (*d* = 9.69). A gene with a smaller Ct value fluctuation range has more stable expression, while a larger Ct value fluctuation range indicates less stable expression. Therefore, the qPCR results showed that the expression stability of *Rps21* and *eIF5A* is the best. The arithmetic mean values of the Ct values for *actin*, *Rps21*, *eIF5A*, *a-tubulin*, *UBI*, *β-PP1* and *18S rRNA* are 22.08, 24.14, 25.43, 29.73, 24.23, 28.55 and 14.51, respectively. The arithmetic mean values of the Ct values of each candidate reference gene in descending order are *18S rRNA*, *actin*, *Rps21*, *UBI*, *eIF5A*, *β-PP1* and *a-tubulin*, indicating that the expression levels from high to low are *18S rRNA*, *actin*, *Rps21*, *UBI*, *eIF5A*, *β-PP1*, *a-tubulin*. The ideal reference gene expression should be neither too high nor too low but should be moderately expressed with a Ct value between 15 and 30 (Lilly et al., 2011; Wan et al., 2010). In the present study, the remaining 6 candidate reference genes, except for *18S rRNA*, which has an arithmetic average of Ct values of only 14.51, are moderately expressed, indicating that *18S rRNA* is the highest expressed among seven candidate reference genes in *R. similis*. However, the overexpression of the reference gene in the quantitative analysis is too large to affect the accuracy of the quantitative results (Vandesompele et al., 2002). Therefore, *18S rRNA* is not an ideal reference gene in this study.

### BestKeeper analysis

The BestKeeper analysis results (Table 6) show both the SD values and the CV values of the seven candidate reference genes in six populations and in four developmental stages of the GJ population. For different populations, the SD values and the CV values of the seven candidate reference genes sorted in ascending order are *Rps21*, *UBI*, *18S rRNA*, *eIF5A*, *actin*, *a-tubulin* and *β-PP1* and *Rps21, UBI, eIF5A*, *a-tubulin*, *β-PP1*, *actin*, and *18S rRNA*, respectively. For different developmental stages of the GJ population, the SD values and the CV values of the seven candidate reference genes sorted in ascending order are *actin*, *eIF5A*, *Rps21*, *β-PP1*, *UBI*, *a-tubulin*, and *18S rRNA* and *actin*, *eIF5A*, *β-PP1*, *Rps21*, *a-tubulin*, *UBI*, and *18S rRNA*, respectively. For different populations, the SD values and the CV values of *Rps21*, *UBI* and *eIF5A* are relatively small among the seven candidate reference genes. Although the SD value of *18S rRNA* is also relatively small, its CV value is the largest among the seven genes. Therefore, *Rps21*, *UBI* and *eIF5A* are relatively stable candidate reference genes. Further analysis shows that the *r* value of *eIF5A* is the largest (0.83), so the BestKeeper program anticipates that the *eIF5A* gene is the most suitable reference gene for different populations of *R. similis*. For different developmental stages, because the SD values and the CV values of *actin*, *eIF5A*, *Rps21* and *β-PP1* are relatively small, these genes can be regarded as suitable reference genes. Further analysis shows that the *r* values of *Rps21* and *eIF5A* are not only the largest two but also very close to each other. Therefore, BestKeeper anticipates that *Rps21* and *eIF5A* are the most suitable reference genes for different developmental stages of one certain population.

**Figure 7:**
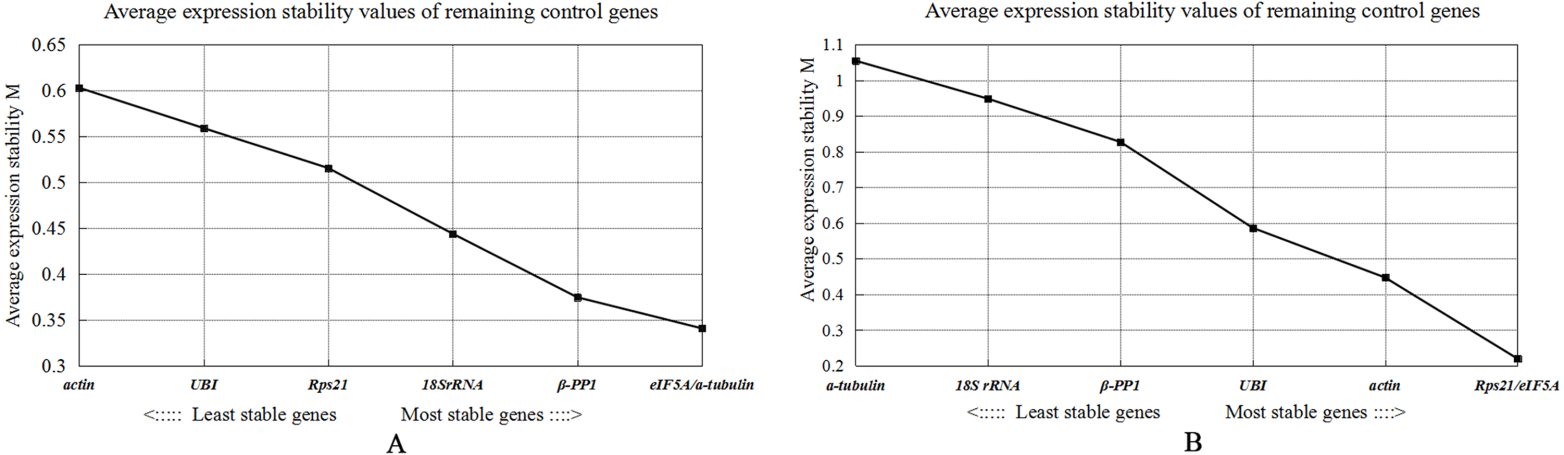
Gene expression stability measures M analysis of seven candidate reference genes under two experimental conditions of *Radopholus similis*. (A) mixed-stage nematodes of six populations of *R. similis* isolated from *Anubias barteri* var. barteri, *Citrus reticulata* Blanco, *Anthurium andraeanum, Musa* AAA Giant Cavendish cv. Baxi, *Zingiber officinale* and *Radix curcumae*. (B) Four developmental stages including female, male, larva and egg of the *R. similis* GJ population isolated from *Citrus reticulata* Blanco.

**Table 6:** BestKeeper analysis results of seven candidate reference genes under two experimental conditions of *Radopholus similis*.

Gene name	Different populations^a^			Different stages^b^		
	SD[±Ct]	CV[%Ct]	*r*	SD[±Ct]	CV[%Ct]	*r*
*actin*	0.56	2.64	0.72	0.36	1.56	0.09
*Rps21*	0.18	0.77	0.51	0.51	2.06	0.81
*eIF5A*	0.46	1.80	0.83	0.45	1.76	0.79
*a-tubulin*	0.6	2.05	0.84	0.86	2.81	0.53
*UBI*	0.29	1.23	0.44	0.83	3.30	0.77
*β-PP1*	0.63	2.40	0.99	0.60	1.86	0.62
*18S rRNA*	0.42	3.07	0.82	1.09	6.92	0.93

**Notes.**

SD Standard Deviation. CV coefficient of variance r coefficient of correlation

a mixed-stage nematodes of six populations of *R. similis* isolated from *Anubias barteri* var. barteri, *Citrus reticulata* Blanco, *Anthurium andraeanum, Musa* AAA Giant Cavendish cv. Baxi, *Zingiber officinale* and *Radix curcumae*.

b Four developmental stages including female, male, larva and egg of the *R. similis* GJ population isolated from *Citrus reticulata* Blanco.

### geNorm analysis

**Figure 8:**
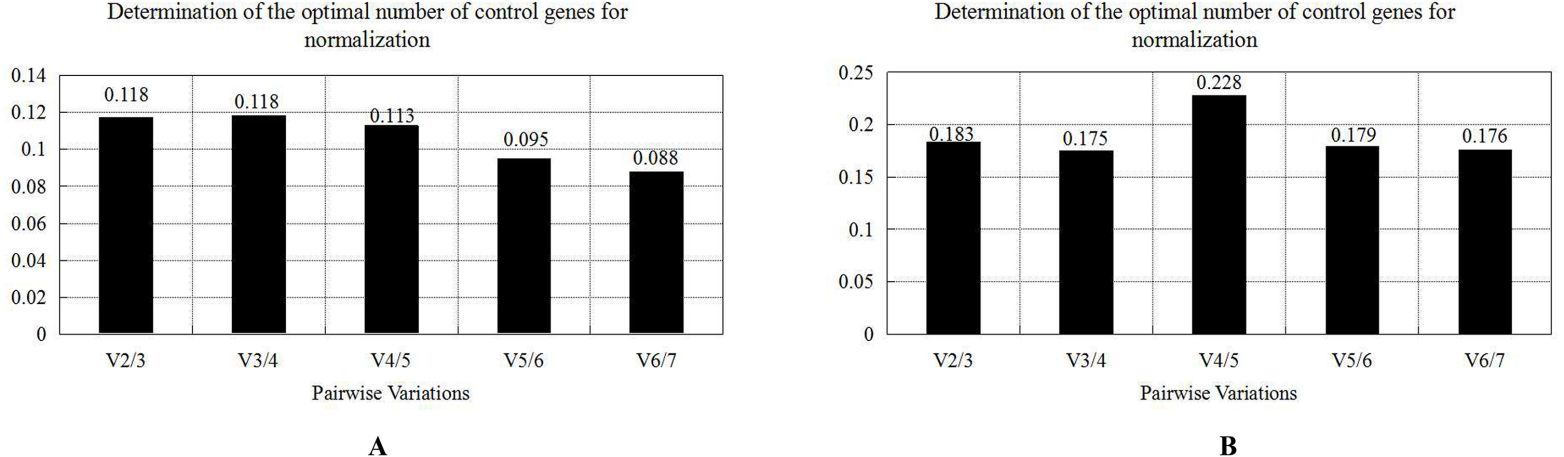
Pairwise variance V analysis of seven candidate reference genes under two experimental conditions of *Radopholus similis*. (A) Mixed-stage nematodes of six populations of *R. similis* isolated from *Anubias barteri* var. barteri, *Citrus reticulata* Blanco, *Anthurium andraeanum, Musa* AAA Giant Cavendish cv. Baxi, *Zingiber officinale* and *Radix curcumae*. (B) Four developmental stages including female, male, larvsolated from *Citrus reticulata* Blanco.

The geNorm analysis results of each candidate reference gene in six populations of *R. similis* and four developmental stages of the *R. similis* GJ population show that the M values of seven candidate reference genes are all less than 1.5, indicating that all seven genes are suitable reference genes. The expression stability of the seven candidate reference genes in both different populations and different developmental stages of the GJ population are sorted in descending order according to the principle that the smaller the M value, the better the expression stability of the gene: *eIF5A/a-tubulin*, *β-PP1*, *18S rRNA*, *Rps21*, *UBI*, and *actin* (Fig. 7A) and *Rps21*/*eIF5A*, *actin*, *UBI*, *β-PP1*, *18S rRNA*, and *a-tubulin* (Fig. 7B), respectively. Pairwise variance V analysis of the seven candidate reference genes showed that V2/3 for different populations =0.118 < 0.15 (Fig. 8A), while all V values for different developmental stages of the GJ population are greater than 0.15 (Fig. 8B). In this case, the default V value for different developmental stages of the GJ population is adjusted to 0.2 according to the needs of this experiment, suggesting that the most suitable number of reference genes for both different populations and different developmental stages of *R. similis* is two. Taking M values into consideration, it can be determined that the most suitable reference genes for different populations of *R. similis* are *eIF5A* and *a-tubulin*, while the most suitable reference genes for different developmental stages of one certain population are *Rps21* and *eIF5A.*

### NormFinder analysis

NormFinder analysis results show both the stability value and the standard error of each candidate gene in different populations and different developmental stages of the GJ population (Table 7). For different populations, not only the stability values of *β-PP1* and *eIF5A* are the lowest but also their standard errors are the lowest among seven candidate reference genes; for different developmental stages, not only the stability values of *eIF5A* and *Rps21* are the lowest but also their standard errors are the lowest among seven candidate reference genes. Therefore, NormFinder anticipates that the most suitable reference genes for different populations of *R. similis* are *β-PP1* and *eIF5A*, and the most suitable reference genes for different developmental stages of one certain population are *eIF5A* and *Rps21*.

**Table 7:** NormFinder analysis results of seven candidate reference genes under two experimental conditions of *Radopholus similis*.

Gene name	Different populations^a^		Different stages^b^	
	Stability value	Standard error	Stability value	Standard error
*actin*	0.0279	0.0094	0.0371	0.0178
*Rps21*	0.0183	0.0069	0.0137	0.0137
*eIF5A*	0.0116	0.0056	0.0135	0.0137
*a-tubulin*	0.0150	0.0062	0.0388	0.0183
*UBI*	0.0228	0.0080	0.0371	0.0178
*β-PP1*	0.0087	0.0055	0.0229	0.0140
*18S rRNA*	0.0250	0.0086	0.0697	0.0293

**Notes.**

a mixed-stage nematodes of six populations of *R. similis* isolated from *Anubias barteri* var. barteri, *Citrus reticulata* Blanco, *Anthurium andraeanum, Musa* AAA Giant Cavendish cv. Baxi, *Zingiber officinale* and *Radix curcumae*.

b Four developmental stages including female, male, larva and egg of the *R. similis* GJ population isolated from *Citrus reticulata* Blanco.

## Discussion

At present, *actin* and *18S rRNA* are the main reference genes in the study of pathogenic genes of *R. similis*. *actin* is an essential cytoskeletal protein that is an important component of cells to maintain basic life activities and plays an important role in cell secretion, phagocytosis, migration, cytoplasmic streaming and cytoplasmic segregation and recombination (Fu et al., 2013). In theory, it can be stably expressed in cell growth and development, suggesting that it might be an ideal reference gene. *18S rRNA* is also a traditional reference gene that is commonly used in plant nematode research. However, this study demonstrated that *actin* and *18S rRNA* are not the best reference genes among the seven candidate reference genes. For the other five candidate reference genes selected in this study, *eIF5A*, *a-tubulin* and *UBI* are conventional reference genes in other organisms, *Rps21* and *β-PP1* are newly identified candidate genes from the transcriptome data of *R. similis*. *eIF5A* is a type of eukaryotic translation initiation factor that promotes the activity of protein synthesis by binding to active ribosomes involved in translation and is involved in the extension of protein translation (Cano et al., 2010; Elfgang et al., 1999; Frigieri et al., 2008; Jao & Chen, 2010; Zanelli et al., 2006). *a-tubulin* is a type of tubulin that exists as a dimer in the cell with *β*-*tubulin* and is involved in important physiological functions such as cell division and differentiation, substance transportation and signal transduction (Wolf & Spanelborowski, 2013). *UBI* (Ubiquitin) is the main part of the ubiquitin-mediated protein degradation pathway that plays an important role in both intracellular degradation of proteins and many basic cellular processes (Pickart, 2001). *β*-*PP1* (serine/threonine protein phosphatases PP1-beta catalytic subunit) is a catalytic subunit of serine/threonine phosphatases (PSPS), which can not only assist the serine/threonine phosphatase in dephosphorylating substrate molecules but also interact with protein kinases to realize the signal cascade and transmission by the phosphorylation and dephosphorylation of substrate molecules (Shi, 2009). *Rps21* (RPS-21 protein) is an important member of the ribosomal protein, and a variety of ribosomal proteins are involved in important processes of ribosome translation, transcriptional regulation, cell development and cell differentiation (Ferguson et al., 2015; Orelle et al., 2015; Takada & Kurisaki, 2015). These genes, similar to *actin*, bear the basic life-function of cells and are theoretically stably expressed in all physiological states of the cells and thus have the potential to become ideal reference genes. Nevertheless, the applicability of these reference genes is not the same for different organisms and for different experimental conditions, which is proven by this study. Blindly using traditional reference genes may yield erroneous results (Livak & Schmittgen, 2001), and ideal reference genes must be screened through experiments.

The results of different software programs in analyzing candidate reference genes are inconsistent in this study. The analyses of BestKeeper, geNorm and NormFinder showed that the most suitable reference gene is *eIF5A*, *eIF5A* and *a-tubulin*, and *β-PP1* and *eIF5A* for six populations of *R. similis*, respectively, while *Rps21* and *eIF5A*, *Rps21* and *eIF5A*, and *Rps21* and *eIF5A* were the best for four developmental stages of one population of *R. similis*, respectively. Therefore, *eIF5A* should be chosen as the reference gene when the experimental objects are different populations of *R. similis*, while *Rps21* and *eIF5A* are both suitable reference genes for different developmental stages in one certain population of *R. similis*. Interestingly, the results of the three software programs for analyzing the seven candidate reference genes in different developmental stages of a population of *R. similis* are consistent, but the results are inconsistent when analyzing different populations. The difference between the results of this analysis is probably due to inconsistencies between different software algorithms. Therefore, to obtain credible results, different software analyses should be used, and their results should be compared to select the most stable and most suitable candidate gene as the reference gene. At the same time, we should pay attention to the applicability of different reference genes under different experimental conditions to ensure the best experimental results.

Ideally, at least ten reference genes should be analyzed for this type of methodological study; however, only seven reference genes were tested in this study, which may cause some limitations to the results of this study.

## Conclusions

In this study, three software programs, BestKeeper, geNorm and NormFinder, were used to analyze the expression stability of seven candidate genes in different populations of *R. similis* and different developmental stages of the GJ population, the results reveal that *eIF5A* is an ideal reference gene in all experimental conditions, indicating *eIF5A* is the most suitable reference gene for use in *R. similis*.

##  Supplemental Information

10.7717/peerj.6253/supp-1 File S1 Raw data including qPCR results, screening results of three software and standard curves

10.7717/peerj.6253/supp-2 Supplemental Information 1 Partial coding sequences of the 6 candidate genes

## Additional Information and Declarations

### DNA Deposition

The following information was supplied regarding the deposition of DNA sequences: The transcriptome data of Radopholus similis here are accessible via GenBank accession numbers SRR6425985–SRR6425988. The candidate reference genes here are accessible via GenBank accession numbers MH499256 to MH499261.

### Data Availability

The following information was supplied regarding data availability: The raw data are available in the Supplemental Files.
