# Supplementary material for: Screening of reference genes in real-time PCR for Radopholus similis
Source: PeerJ. 2019 Jan 16;7:e6253. doi: 10.7717/peerj.6253 (PMC6339476; doi:10.7717/peerj.6253)
Supplement: Supplemental Information 1 [file peerj-07-6253-s002.doc]

>Seq1 [organism=Radopholus similis] **actin** mRNA, partial cds (MH499256,382bp)

ATGGTCGGAATGGGACAGAAGGACTCCTACGTCGGAGACGAGGCGCAGTCCAAGCGTGGTATCCTGACCCTGAAGTACCCGATTGAGCACGGCATCGTGACCAACTGGGACGACATGGAGAAGATCTGGCACCACACCTTCTACAACGAGCTGCGTGTCGCCCCCGAGGAGCACCCAGTGCTGCTGACCGAGGCGCCGCTGAACCCGAAGGCCAACCGTGAAAAGATGACCCAGATCATGTTCGAGACCTTCAACACGCCCGCCATGTACGTCGCCATCCAGGCCGTGCTGTCGCTGTACGCCTCCGGACGTACCACCGGCATTGTGCTCGACTCTGGCGATGGTGTGACCCACACCGTGCCCATCTACGAGGGTTACGCCC

>Seq2 [organism=Radopholus similis] Ribosomal protein S21 **(Rps21)** mRNA, partial cds (MH499257,344bp)

ACCCAGTACTCAAGGTCAAATCTTTCTAAAGTTGCACGCACGTTAAAAATGCAGAACGACGCCGGCGAACTTGTGGAGCTGTATGTTCCTCGCAAATGCTCTTCATCTTCGCGGATCATCGCCGCTAAAGACCACGCCTCGATCCAGCTAGACATTGTTGGCGTAAACCCGGAAACCGGTCGTATGGACCCGAGCAAAACGACCAAATATGCGATCTGTGGAAAGCTCCGTTTCATGGGCGAGTCCGACGATTGCATCCTGCGTTTGGCACAGAAAGATGGAATTGTTCCAGAAAAGATGTAATCGTCTGCGCGATCCAAAATTGCGATTTGTTGACTGTTTCT

>Seq3 [organism=Radopholus similis] Eukaryotic translation initiation factor 5A **(eIF5A)** mRNA, partial cds (MH499258,556bp)

GCCGCTGCCACTTACCCGAAACAATGTTCGGCCTTGCGCAAAAACGAATTCGTTATGATCAAGGGACGTCCGTGCAAGGTTGTAGAGATGAGTACTTCGAAGACCGGTAAACACGGGCATGCAAAGGTGCACCTGGTGGCGCTCGATATCTTCACTAATAAGAAGCTTGAAGACATTTGCCCTTCCACCCACAATATGGAAGTGCCGGTTGTGAAGCGCAAGGAGTACCAGCTTCTTTCTGTCAATGAGGACGGTTTTGTCAGCATGATGGATTTGGAGAGCTGCGACACCAAGGATGACTTGCGTTTACCAGAGGGTGAGATTGGGGACCAGATAAAACAGGCGTACGAGAAAGACGAGAACGGCATACTTGTCAATGTGGTGTCCGCTTGCGGCGAGGAAGCTATTCACGGGTGGAAGTACATGCCAAATCGCGAATAATTGTTGCCCACGGCAGGCACGGGAGAGACGAGGATGAGTTTGGGTGAAAATGGAGGGAGGATGGTCGACGTAAAAGACAGCATTCGATCAACAAATTCTCCTCTCTCTCGTCCAA

>Seq4 [organism=Radopholus similis] Tubulin alpha **(a-tubulin)** mRNA, partial cds (MH499259,538bp)

ATCACCGCATCTCTCCGCTTCGACGGCGCACTCAACGTGGACCTGACGGAGTTCCAGACCAATCTGGTCCCCTACCCACGCATCCATTTCCCGCTGGCGACCTATTCGCCTATCATTTCCGCTGAACGTGCGTTCCACGAGCACATGTCAGTGCCAGAAATCACAAACAAATGCTTCGAGGCAGGCCATCAGATGGTCAAGTGCGACCCGCGCAACGGAAAATACATGGCATGTTGCTTGTTGTTCCGTGGCGACGTGGTCCCGAAGGACGTGAACGCGGCGATTGCGACGGTGAAGACCAAACGGGCTATCCAGTTCGTGGACTGGTGTCCGACCGGCTTCAAAGTGGGCATCAACTACCAGCCGCCAACTGTGGTTCCCGGAGGGGATTTGGCCAAACTGCAGAGAGCCGTCTGCATGCTGTCCAACACAACCGCGATTGCGGAGGCATGGACCCGTCTGGACCACAAGTTCGATTTGATGTACTCAAAGCGTGCATTTGTGCACTGGTACGTCGGAGAGGGAATGGAGGAAGGCG

>Seq5 [organism=Radopholus similis] Ubiquitin protein **(UBI)** mRNA, partial cds (MH499260,473bp)

CGTGAAAACTCTGACTGGAAAGACCATCACTCTGGAAGTGGAGGGATCGGACACCATCGAGAACGTCAAGGCCAAGATCCAGGACAAGGAGGGCATCCCGCCCGACCAACAGCGATTGATCTTCGCCGGGAAACAGCTCGAAGACGGTCGCACGCTTGCCGACTACAACATTCAAAAGGAGTCCACTCTCCATTTGGTGTTGCGTCTCCGCGGTGGAATGCAGATCTTCGTCAAGACCCTCACCGGAAAGACCATCACTCTCGAAGTCGAGGGTTCGGACACCATCGAGAATGTGAAGGCCAAGATCCAGGACAAGGAGGGCATCCCGCCCGACCAACAGCGTCTGATCTTCGCCGGGAAACAACTTGAGGACGGTCGCACTCTTGCGGACTACAACATCCAGAAGGAGTCCACTCTCCATCTGGTGCTGCGTCTCCGCGGTGGTTCCGCCTGAATGGGAGAAAGCGCAGAGG

>Seq6 [organism=Radopholus similis] serine/threonine protein phosphatase PP1-beta catalytic subunit **(β-PP1)** mRNA, partial cds (MH499261,719bp)

CTTCTGCTGTCATGGCGGACTTTCGCCGGATCTACAAAACATGGAACAGTTCCGCAGGATCATGCGGCCGACGGACGTACCTGATACTGGTTTGTTATGCGACCTTCTGTGGTCGGATCCCGACAAAGATGTCCAAGGCTGGGGAGAAAACGATCGTGGTGTCAGTTTCACATTCGGGCCAGACGTTGTGGGAAAGTTTCTGAACCGACACGACCTGGACCTGATATGTCGGGCACATCAGGTTGTTGAGGACGGGTACGAATTTTTTGCAAAGCGCCAACTGGTTACACTCTTCTCCGCGCCAAATTACTGTGGAGAGTTCGATAATGCTGGCGGCATGATGTCTGTCGATGAAACACTCATGTGCTCGTTCCAGATCCTGAAACCATCCGAGAAGAAGGCGAAGTACCAGTACGCCGGTCTGAACAGCGGCAGGCCTTCGCAATCACCCGTCCAACGCAATCAGCAACCGCCAGGTGTGCAGGGCAAAAAAAAATGACAAAGACAATTGATGCGGTAAATCGGATGCCTACTTGCTTAAACTGAAAACCCTCCATTTATAATTGTTTTTATGCATGGCGATGATTTGACTTTTTAATCGTGAGATGGGCGAATTCATGGCAATATATCGAGGGATGATTTTAATGTTTCTTTACCGTCGGCCTACGATCCCGATAACGCTGATCAAAAATTGAAAGGTTAGCAGCACTACGACCGTC
